# Stacking Interactions of Poly Para-Phenylene Vinylene Oligomers with Graphene and Single-Walled Carbon Nanotubes: A Molecular Dynamics Approach

**DOI:** 10.3390/molecules25204812

**Published:** 2020-10-20

**Authors:** Nii Amah Dagadu, Shahram Ajori, Yaw Delali Bensah, Kwabena Kan-Dapaah, Stephen Kofi Armah, Boateng Onwona-Agyeman, Abu Yaya

**Affiliations:** 1 Department of Materials Science and Engineering, School of Engineering Sciences, CBAS, University of Ghana, Legon P.O. Box LG 77, Ghana; niidagadu@gmail.com (N.A.D.); ydbensah@ug.edu.gh (Y.D.B.); skarmah@ug.edu.gh (S.K.A.); BOnwona-Agyeman@ug.edu.gh (B.O.-A.); 2 Department of Mechanical Engineering, Faculty of Engineering, University of Maragheh, Maragheh P.O. Box 55136-553, Iran; sajori@maragheh.ac.ir; 3 Department of Biomedical Engineering, School of Engineering Sciences, CBAS, University of Ghana, Legon P.O. Box LG 77, Ghana; KKan-Dapaah@ug.edu.gh

**Keywords:** poly para-phenylene vinylene (PPV) oligomers, graphene, carbon nanotube, interfacial interaction

## Abstract

This study is meant to address the understanding of the interactions between poly para-phenylene vinylene (PPV) oligomers, graphene and single-walled carbon nanotubes (SWCNT). To this end, the binding energies of the PPV oligomers with graphene and SWCNTs were investigated. Calculations are performed and the parameters related to van der Waal vdW interactions are discussed to achieve and confirm the crystallization of oligomers of PPV into herringbone (HB) structure arrangement, which is known to be the most stable conformation at 300 K. Finally, the interfacial interactions between crystal PPV, graphene and SWCNT are carried out. According to the results, the intramolecular potential energies of PPV chains are found to increase linearly with each extending PPV monomer unit by approximately 50 kcal/mol. Moreover, the interfacial interaction properties analysis using radial distribution functions (RDFs) for PPV-graphene and PPV-SWCNT show significant disordering of the arrangement of molecules, which is more pronounced for PPV-SWCNT than that in PPV-graphene. The radius of gyration ($R_{g}$) profiles show a net decrease of ∼−0.8, for PPV-graphene with different surface coverage, and, a net increase of ∼+0.6, for PPV-SWCNT; meaning that, the binding between PPV-graphene is much stronger than with PPV-SWCNT.

## 1. Introduction

Nanomaterials are presently a major focus in materials science & engineering. The ability to uniquely tailor their electrical, thermal, and mechanical properties amongst others through doping and stacking configurations has demonstrated their importance in engineering properties of bulk materials. Their understanding and development are crucial and makes them an attractive approach to improve the properties of classical materials, and the design of new ones. The potential applications of these nanomaterials are broad, and span across almost every industry. For example, intrinsically conducting polymers (ICPs) have become a significant aspect in the continuation of Moore’s law in the electronic industry, i.e., the number of transistors on integrated circuits roughly doubles biennially [1]. The reason for this is that the organic molecules can be easily processed, and uniquely adapted through doping in order to surpass the limitations of contemporary metal-oxide devices [2]. Electrical devices such as organic light-emitting diodes (OLEDs) [3], organic field-effect transistors (OFETs) [4], photovoltaic cells (PVCs) [5], light amplification by stimulated emission of radiation (LASER) devices [6] and sensors [7] have been manufactured from conducting polymers, and as such, significant resources have been invested in their research. Moreover, oligomers of conducting polymers have been proven to be an alternative to longer-chain polymeric systems. They require less effort to be functionalized and produced in large quantities and their synthesis can be well controlled to achieve high quality. This is compounded by the fact that oligomeric systems possess similar physical attributes to longer-chain systems such as the molecular arrangement, density, and conductivity [2,8], and thus, can be reliably used in characterization and substitution of larger systems.

Poly (para-phenylene-vinylene), (PPV), is an organic polymer that is semiconducting [9] and possesses high crystallinity. It is currently used in PVCs [10], OLEDs [11] and OFETs [4]. Potential applications in laser technologies [12,13], and in bio-imaging [14], have also been reported. Characterizing and controlling the properties of PPV oligomers, in order to improve the performance and efficiency of application has been receiving a lot of research attention in the scientific community and industry. Furthermore, PPV oligomers and derivatives have been shown to be efficient in multiple applications [15,16,17,18,19].

**Figure 1:**
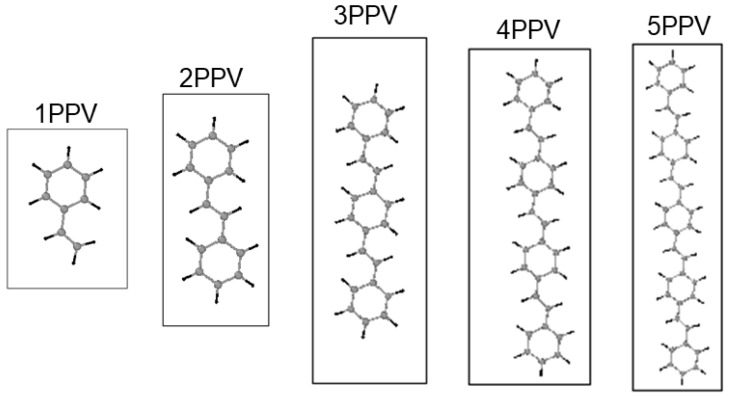
Poly (para-phenylene-vinylene) (PPV) oligomers and their designated names in this work.

Carbon nanomaterials (CNMs), such as graphene and carbon nanotubes (SWCNTs) have very attractive engineering properties, which include high conductivity, thermal stability, light-weight, ultra-strength, and covalent doping capabilities, which have made them such promising candidates for applications in nanoelectromechanical systems (NEMS). In some organic solar devices, CNMs perform as acceptors while conducting polymers operate as the donors [20]. Previous studies indicate that incorporating CNMs into nanocomposites improves the conductivity, thermal stability, and strength of the nanocomposite [21,22,23]. For example, pristine graphene, which has no band gap, can be covalently doped into a semiconductor by polymers for potential transistor gate applications [24]. Additionally, Guo et al., [25] reported a significant band gap closing in semiconducting SWCNT to a semi-metallic state upon interaction with distyrylbenzene (an oligomer of PPV, three-PPV oligomer chains (3PPV or $P_{3}V_{2}$) and an upward shift in energy levels of SWCNTs, see Figure 1. It is essential to investigate (post) crystallization interfacial effects of CNMs with its polymer matrix as this influences the electronic states of nearby atoms and molecules.

The physical configuration at interfaces is of importance as the arrangements of vicinity particles dictates how much the electron densities (or distributions) interact, which has a direct consequence on several bulk properties of the nanocomposite. It was observed that the organic-based electronics possess better and amenable properties, resulting in more potential applications than silicon-based electronics [1]. It was shown that interfaces of some organic devices become misaligned, and that substrate choice affects the final structure [26,27,28]. Therefore, making the case that efficiency, effectiveness, and lifespan of such devices are dictated by interaction energy and interfacial conformation.

Additionally, there are challenges in predicting the end assembly/configuration of the molecule, which poses a problem when regulating the manufacture and industrial production of the materials. This results in variations in batch quality and performance. In some cases, small defects (sometimes much less than 1%) have a large impact on intended application performances. Experimental [11,26,29] and ab initio (quantum mechanical) [9,30,31] studies in literature aim at understanding electronic and structural properties of materials such as PPV and CNMs, and their interactions. Quantum mechanics provides highly accurate solutions that possess some drawbacks to the inherent accuracy of ab initio simulations such as restricting the calculations at ground state and computational costs including simulation time and hardware. Classical molecular dynamics present an alternative to study these properties. However, it lacks the high accuracy of ab initio studies due to the lack of accountability of electronic effects. Fortunately, several developed potential force fields allow insight into bulk material configuration and thermodynamic influences on application properties that are not possible in ab initio systems. Therefore, this work is geared towards understanding the interactions between PPV, graphene, and SWCNT using molecular dynamics simulation approach at room temperature (~300 K). This model will be used to confirm the herringbone (HB) arrangement of weak C-H…π interactions, which are known to be the most stable configuration for pristine PPV at 300 K. Furthermore, the interfacial properties between crystal PPV having the herringbone structure together with graphene and SWCNT were investigated and compared. This can be used as a benchmark for further analysis and application of carbon nanomaterial (CNM)-based PPV nanocomposites.

## 2. Materials and Methods

### 2.1. Simulation Method

All simulations have been carried out using Large-Scale Atomic/Molecular Massively Parallel Simulator (LAMMPS) software package [32]. Accordingly, universal force field (UFF) is used for modeling the energetics and interactions of systems [33]. Force switching (fsw) and shifting (fsh) functions were applied to the 6–12 Lennard-Jones potential (LJ) and columbic interactions, respectively [34,35,36]. It is important to note here that due to the large variations of LJ σ and ϵ pairs that have been reported in the literature for C and H, a comparative study between pairs from different sources on the PPV monomer is performed. The Lorentz–Berthelot approach gives the arithmetic and geometric averages between pair interaction of non-identical atoms, Equations (1) and (2), respectively.

(1) $$\sigma_{\mathrm{ij}}=\frac{\sigma_{i}+\sigma_{j}}{2}$$

(2) $$\epsilon_{\mathrm{ij}}=\sqrt{\epsilon_{i}\epsilon_{j}}$$

The pairs are identified by their origins: (i) experimental, and (ii) theoretical. For simplicity, LJ parameters from mixed experimental and ab-initio works were classified as theoretical. The best performing parameter set is used in all subsequent simulations. The visual stability of the trajectories of the molecule (with their periodic images) in capturing the setting angle, $\phi_{s}$ during the final equilibration phase was used as judging criteria where $\phi_{s}$ is defined by the angle between the plane of the PPV molecule and the horizontal plane. The performance of a set of LJ parameters for the monomer did not necessarily transfer effectively to the oligomer systems with the chosen annealing regime. This was because the oligomers contained more atoms per simulation cell, and, therefore, more degrees of freedom.

Thereafter, all simulations were regulated with Nose–Hoover algorithms for thermostats and barostats [37,38,39,40,41], and velocity-Verlet time integration scheme was applied to the equations of motion. A fixed time step of 1 fs was used for all simulations with a cut off distance of  10 Å. A periodic boundary condition was imposed on all crystallization simulations and shrink-wrap boundary conditions were applied to all isolated systems. It should be noted that shrink boundaries are non-periodic boundary conditions (BCs) in LAMMPS that isolate the system and expand or shrink accordingly to prevent particle loss, simultaneously. The tested difference in the potential energies with respect to the application of a large vacuum around the specimen was much less than one percent. Moreover, geometric optimizations were performed with universal force fields (UFFs) as implemented in Arguslab [42,43,44,45,46,47,48,49,50]. The Hamiltonian for the energy expression was evaluated using a Broyden–Fletcher–Goldfarb–Shanno (BFGS) non-linear line search minimization algorithm with a gradient of convergence of $10^{-1}{\;\mathrm{kcal}\;\mathrm{mol}}^{-1}{Å}^{-1}.$ Weak interactions were calculated with a cut-off range between  8–10 Å.

For the binding energies, the systems were equilibrated for 50 ps, followed by a 50 ps production run. Final potential energies were averaged over the production run. The sampling of the ensemble was performed every 0.1 ps. This approach follows closely the methodology employed by Ajori et al. [51,52] and Mirabbaszadeh et al. [53]. To account for crystallization, the systems were first equilibrated at 550 K for 100 ps, then cooled to 300 K for 100 ps, and equilibrated again at 300 K for 100 ps. Finally, the interfacial interaction between HB-PPV, graphene and SWCNT was calculated at 300 K.

### 2.2. Simulation Models

Oligomers were placed within 10 Å of the CNMs. After the energy minimization, canonical ensemble (NVT) was applied to the isolated systems, while isobaric-isothermal ensemble (NPT) with zero pressure was implemented in crystal systems to allow the volume of the system to readjust. System properties were averaged over the final equilibration run. Furthermore, one-PPV chain 1PPV was assumed to be in liquid form at 300 K and therefore its crystallization was not studied. Simulation time for which the generated supercells of PPV were stable without CNM interactions was determined to be approximately 20 ps to calculate the interaction energy by Equation (3).

(3) $$E_{\mathrm{interaction}}\;={\;E}_{\mathrm{CNT}/\mathrm{PPV}}-{\;E}_{\mathrm{PPV}}\;-{\;E}_{\mathrm{SWCNT}}$$

Where ${\;E}_{\mathrm{CNT}/\mathrm{PPV}}$, $E_{\mathrm{PPV}}$, and $E_{\mathrm{SWCNT}}$ are the potential energies of the system, the isolated PPV, and isolated SWCNT, respectively. Moreover, PPV oligomers were ${\;P}_{1}V_{1},{\;P}_{2}V_{1},{\;P}_{3}V_{2},{\;P}_{4}V_{3},{\;P}_{5}V_{4}$, these are termed as 1PPV, two-PPV oligomer chains (2PPV )(also known as (trans-) stilbene), 3PPV, four & five-PPV oligomer chains (4PPV and 5PPV) in this study, respectively, as illustrated in Figure 1.

**Figure 2:**
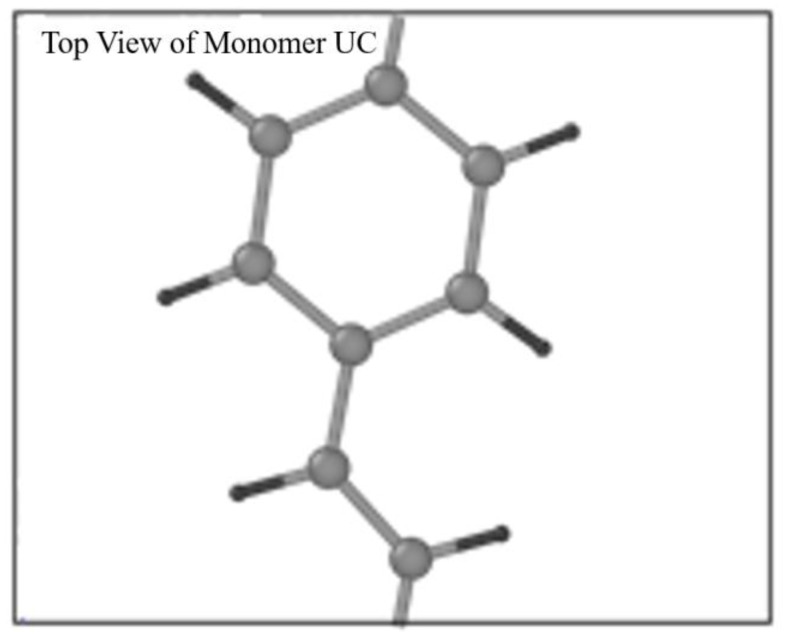
Top view of PPV monomer in unit cell.

**Figure 3:**
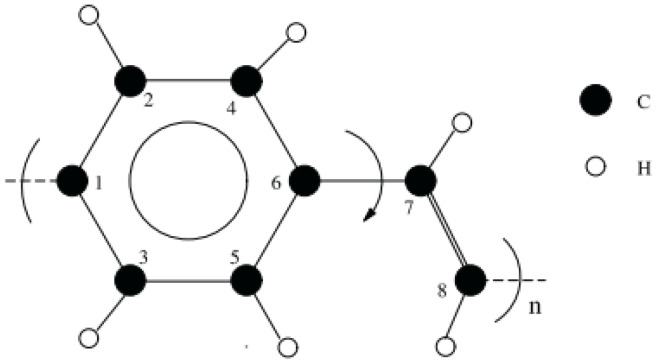
Isolated PPV chain monomer. Numbering is adapted from references [9,54].

The open bonds of the monomer were linked across the periodic boundaries to obtain an “infinitely” continuous polymer chain, as shown in Figure 2. This was done correctly through inspection of the stiffness parameters for longer oligomers that have been optimized (namely 2PPV, 3PPV and 4PPV). Moreover, Figure 3 illustrates the correct labeling of atoms in the monomer used in the validation of the optimized geometry following Capaz et al. [54] and Zheng [9].

A 7 nm×7 nm graphene sheet was used in the simulations for the interaction energies with the oligomers. The dimensions were chosen such that the largest PPV molecules were at least a factor of 2.0 times that of the pairwise interaction cut off distance away from the hydrogen atoms at the edge of the sheet. Consequently, this eliminated or minimized unwanted steric interactions between the molecule and the hydrogen at the edges of the sheet, which would influence the results. In addition, metallic and semiconductor SWCNT with approximate diameters of 4, 6, 8 and 10 Å were selected with the geometrical parameters presented in Table 1. The metallic SWCNTs have a length of $l_{m}=71.4\;Å$, and the semiconductor SWCNTs have a length of $l_{s}=72.5\;Å.$ The same separation factor of 2.0 times the cut-off distance was used, as in the case for the graphene sheets.

## 3. Results

### 3.1. Geometry Optimization

Van der Waal interactions are the major contributors to the final geometry during energy minimizations, with little contributions from the bonds and angles. For example, 12.080 kcal/mol out of the final energy of 15.894 kcal/mol of the styrene, i.e., 1PPV, is from van der Waals interactions; and only 1.833 kcal/mol and 1.981 kcal/mol are from the bonds and angular interactions while there are no considerable contributions from dihedrals, improper and coulombic interactions. The optimized bond lengths and angles for the PPV oligomers were compared to experimental and theoretical values reported in the literature (see Table 2).

Theoretical data [9,54], from Table 2, were obtained with ab initio simulation, while experimental data [55] were obtained through X-ray measurements. There are various non-bonding interaction parameters that exist in the literature. PPV oligomers at 300 K would form the herringbone (HB) structures 300k. Achieving HB structure is due to van der Waals dispersion forces and consequently, it was crucial to obtain the necessary parameters that will result in an HB structure. Obviously, this was not the case for all LJ potentials whether quantum mechanics or molecular mechanics (QM or MM). It turns out different LJ parameters in literature give varying results. Therefore, the simulations have been performed considering provided parameters to reach the most stable parameter for HB. To this end, six LJ parameters were studied: AMBER94 [56], AM1/TIP3P [57], HF/3-21G/TIP3P [58], UFF [47], B3LYP/6-31+G*/AMBER [59] and AUTODOCK [60]. These are henceforth to referred as $LJ_{AMBER}$, $LJ_{AM1}$, $LJ_{HF}$, $LJ_{UFF}$, $LJ_{B3LYP}$ and $LJ_{AUTODOCK}$, respectively. They were classified into two groups: experimental and theoretical. Parameters obtained from combined experimental and theoretical work were classified as theoretical. The corresponding σ and ϵ values are presented in Table 3.

**Figure 4:**
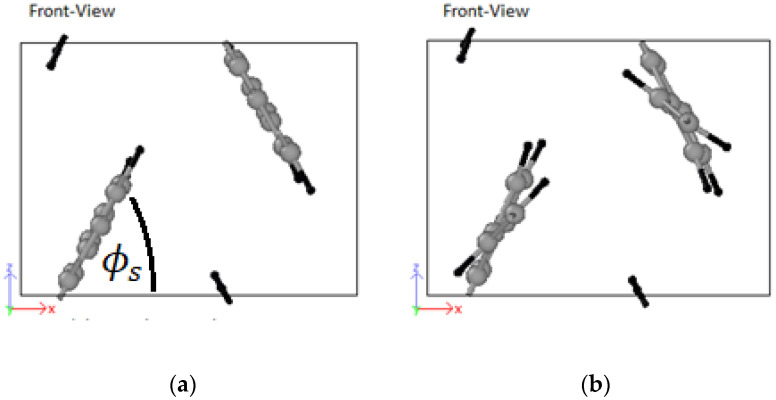
(**a**) PPV molecule with minimal rotation of vinyl link using B3LYP/6-31+G*/AMBER; (**b**) PPV molecule using universal force field (UFF).

It can be observed from Figure 4 that the experimentally obtained σ and ϵ pairs resulted in pronounced out-of-plane rotation of vinyl link with respect to the plane of the PPV molecule (UFF) compared to the theoretically calculated LJ parameters (i.e., $LJ_{B3LYP}$).

**Figure 5:**
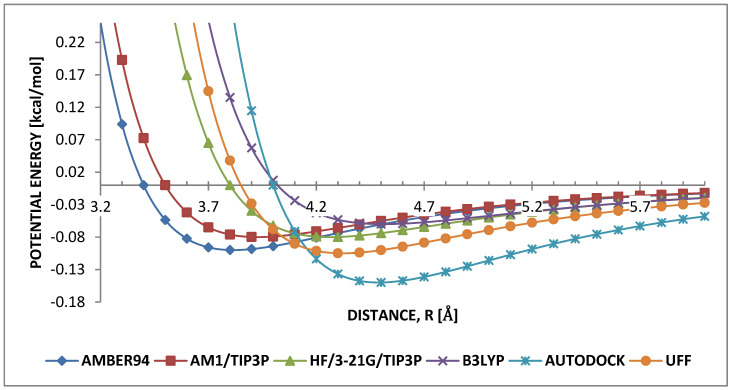
Comparison of Lennard-Jones (LJ) parameters for carbon–carbon.

Two theoretical terms, $LJ_{B3LYP}$ [59] and $LJ_{AUTODOCK}$ [60] gave the most stable HB structure by annealing the monomer from 850 K to 300 K. At higher temperatures the parameters of the remaining terms exhibited unstable non-uniform HB structures, which disappeared into brick-wall shapes upon cooling to 300 K, irrespective of the applied cooling rate. Hence, these were unsuitable for modeling weak C-H…π interactions of PPV. Consequently, the focus of further discussion is based on the values and behavior of well depth ϵ and finite distance of σ regarding B3LYP and AUTODOCK. To achieve a physical insight to this conclusion, carbon–carbon (C…C), hydrogen–hydrogen (H…H) and carbon–hydrogen (C…H) interactions between PPVs were discussed. Considering C…C interaction, Figure 5 shows that $LJ_{B3LYP}$ and $LJ_{AUTODOCK}$ have very high σ values, $\sigma_{B3\mathrm{LPY}}\approx \sigma_{\mathrm{AUTODOCK}}\approx 4$, but significantly different well depths, $\epsilon_{B3\mathrm{LYP}}\approx 0.042$ and $\epsilon_{\mathrm{AUTODOCK}}\approx 0.15$, [59,60]. This suggests that for HB arrangements in PPV, the van der Waals radius of the carbon is more important than the strength of its relative attraction. Consequently, this also contributes to a larger van der Waals radius for the weak carbon to hydrogen interaction.

**Figure 6:**
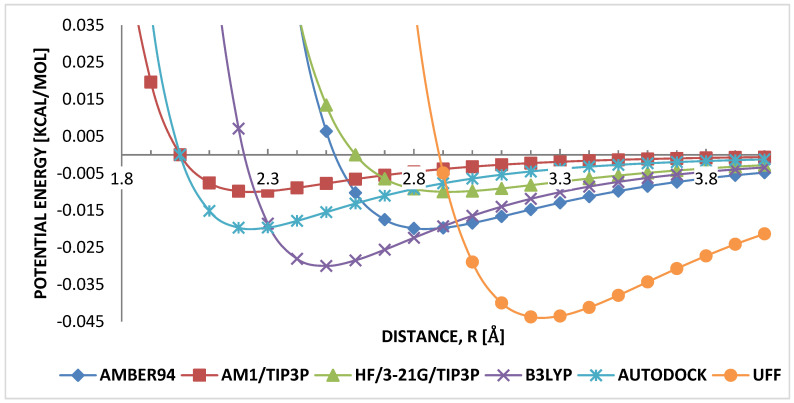
Comparison of LJ parameters hydrogen–hydrogen.

Figure 6 shows the potential profiles for $LJ_{B3LYP}$ and $LJ_{AUTODOCK}$ parameters. They ranked as having the smallest interatomic equilibrium distance, $\sigma_{B3LYP}\approx 2.2\;Å$ and $\sigma_{AUTODOCK}\approx 2.0\;Å$. It is noted that, despite AM1 having the lowest H...H equilibrium distance, considering B3LYP and AUTODOCK, AUTODOCK equilibrium distance is approximately equal to that of AM1, then followed directly by B3LYP parameter. The strength of attraction is comparatively similar, $\epsilon_{B3LYP}\approx 0.02$ and $\epsilon_{AUTODOCK}\approx 0.03$. This similarity in both σ and ϵ parameters indicates that the range of both parameters is important in weak-interacting hydrogen–hydrogen in HB-PPV.

**Figure 7:**
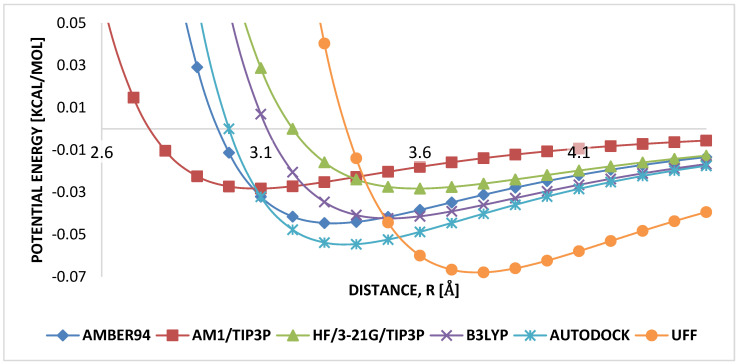
Comparison of LJ parameters carbon–hydrogen.

Weak carbon–hydrogen interactions are governed by the arithmetic mean for σ, and the geometric mean for ϵ. It is seen that $LJ_{B3LYP}$ and $LJ_{AUTODOCK}$ had similar mean parameters for weak interacting carbon–hydrogen, σ≈3.0 Å and ϵ≈0.05, Figure 7, therefore, shows the range where both parameters are independent of each other in the weak carbon–hydrogen interactions in HB-PPV.

### 3.2. Crystallization

**Figure 8:**
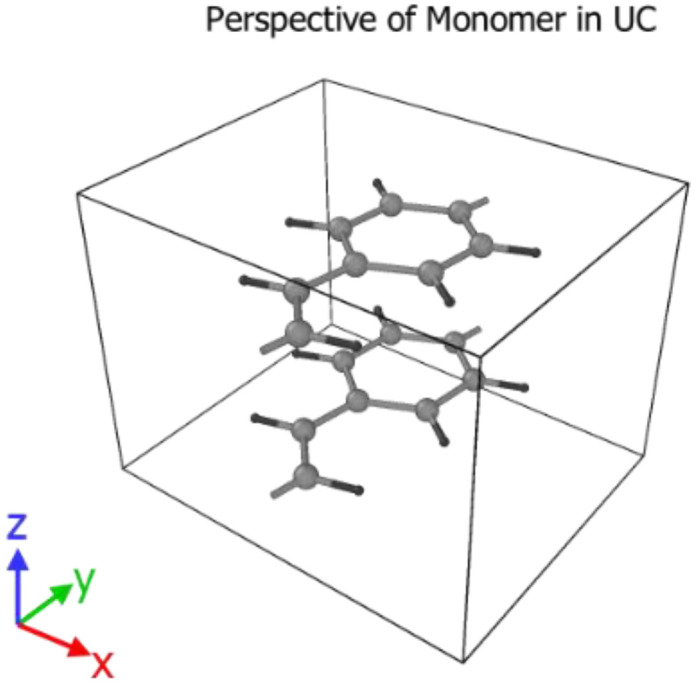
Reference frame and starting orientation of two PPV monomers in the simulation box.

It is crucial to have the unit cell dimensions in the ab- or zx-plane very close to their optimized values as shown in Table 4. The annealing process will otherwise most likely settle in a local minimum, with varying degrees of HB and brick-wall conformations. All molecules were initially parallel to each other in the z-direction (Figure 8).

**Figure 9:**
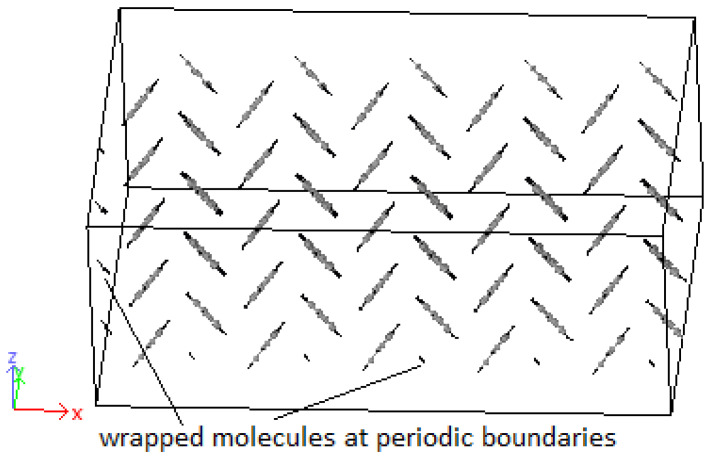
Supercell with wrapped molecules at periodic boundaries.

Crystals were produced by replicating unit cells into supercells (SCs) and optimized to achieve the HB structure. Due to periodic boundaries, molecules at the boundaries may wrap across the boundary depicted in Figure 9. An initial 8 × 1 × 8 supercell of 4PPV is generated. Upon removal of wrapped molecules, a 6 × 1 × 5 supercell remained. Crystals of 4PPV were used in interfacial simulations as 3PPV and 5PPV behave similarly.

**Figure 10:**
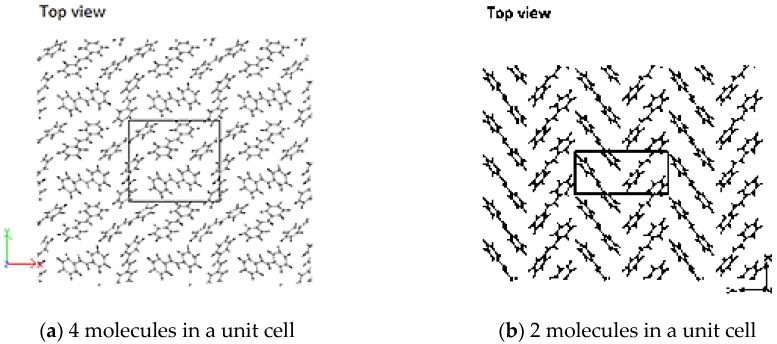
(**a**) Stilbene using unit cell by [55]; (**b**) herringbone- (HB)-packing of 2PPV (stilbene) from simulation using 2 molecules.

The initial concentration/density of molecules in the simulation cell affected the conformation of the resulting crystal structure. Sparsely populated simulation cells have more space to freely orient, and in some cases, there is re-ordering to other HB-structures, similar to that observed in trans-stilbene crystals. This highlights the fact that HB-structured PPV is of a densely packed nature; and perhaps, as well, hints to other possible conformations of the oligomers dependent on concentrations [17]. Simulation of the unit cell with four molecules as specified by Finder et al. [55] resulted in an HB structure with a high degree of misalignment (Figure 10a). Constraining the simulation cell to a=Δx=6.2 Å, b=Δz=6.0 Å and c=Δy=14.4 Å, where Δx≈12.381/2 (half of that in reference [55]) with only two molecules of 2PPV results in a proper HB arrangement similar to that specified by Finder et al. (Figure 10b). In doing so, the reduction in the initial ab-plane cross-section of the unit cell results in the reduction in its volume as well. This shows how the number of interacting molecules and the ab-plane cross-section of the simulated unit cell dictate the final structure.

**Figure 11:**
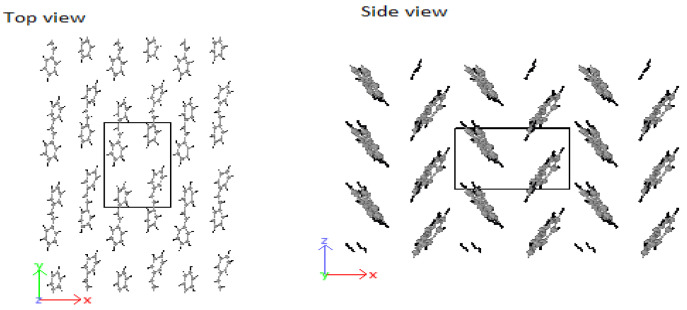
HB-structure of stilbene at a=Δx=8.07 Å and b=Δz=6.05 Å.

Based on the above discussions, stilbene was simulated with an ab-plane cross-section of a=Δx=8.07 Å and b=Δz=6.05 Å. The result was an HB-structure as obtained for the higher oligo-PPV molecules, as seen in Figure 11.

**Figure 12:**
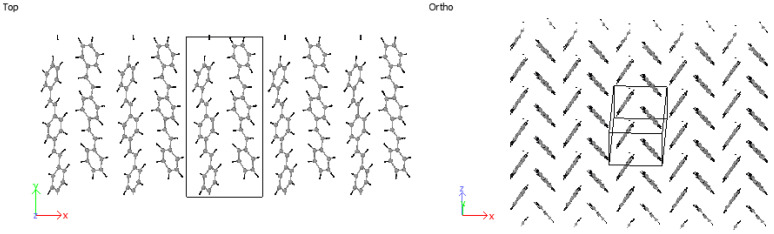
Three-PPV oligomer chains (3PPV) HB-packing from simulation.

**Figure 13:**
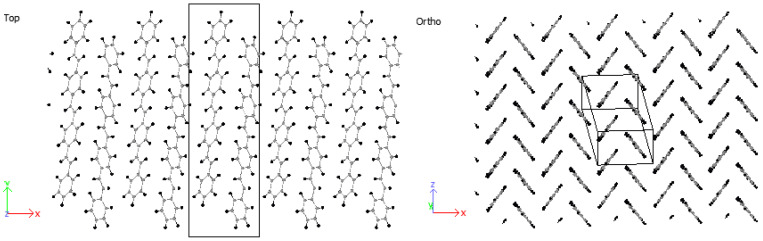
Four-PPV oligomer chains (4PPV) HB-packing from simulation.

**Figure 14:**
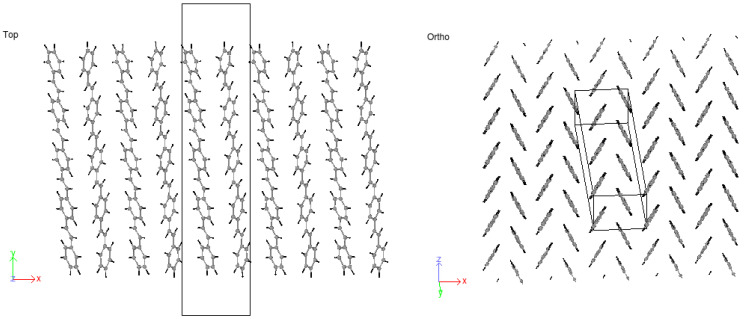
5PPV HB-packing from simulation.

Considering 3PPV in Figure 12, the unit cell is of the dimensions a=Δx = 8.89 Å, b=Δz = 5.92 Å and c=Δy = 18.76 Å. The Δy dimension is roughly three times the length of the monomer unit cell c=6.54 Å≈Δy/3 and the setting angle $\;\phi_{s}∼\;60^{{}^{\circ}}$. It should be noted that all dimensions are comparable to the monomer crystal. Consequently, HB arrangements are obtained for 4PPV and 5PPV under simulation conditions as specified in the methodology, Figure 13 and Figure 14, respectively.

### 3.3. Intramolecular Interactions and Binding Energy

**Figure 15:**
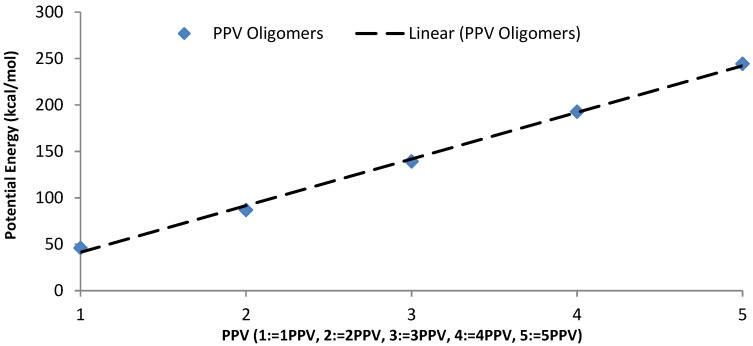
Intramolecular potential energy of PPV oligomers.

The intramolecular energies of the isolated oligomers are presented in Figure 15. As expected, the potential energy of 1PPV, 2PPV, 3PPV, 4PPV and 5PPV, increased linearly by approximately 50 kcal/mol, corresponding directly to the increase in consecutive chains by one monomer unit each.

**Figure 16:**
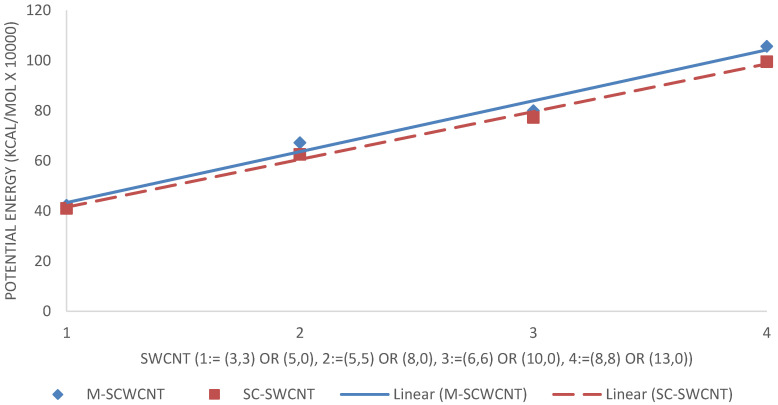
Intramolecular potential energy of SWCNTs.

The potential energy of the 7 nm×7 nm hydrogen-terminated graphene sheet was obtained as 2,191,330 kcal/mol. Considering the SWCNTs shown in Figure 16, SWCNTs with larger diameters contain more atoms, and therefore, have more energy. Metallic SWCNTs (M-SWCNTs) have slightly more potential energy than semiconducting SWCNTs (SC-SWCNTs), this was to be expected as the diameters of metallic SWCNT are fractionally larger, and hence they contain more atoms.

**Figure 17:**
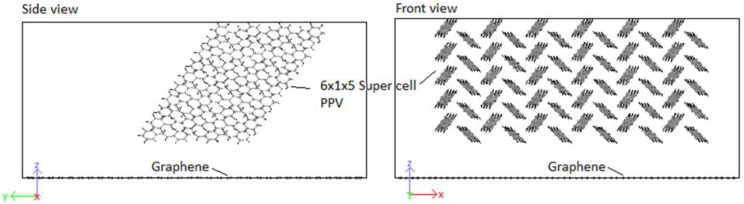
Initial configuration of 4PPV–7 nm × 7 nm graphene interaction.

**Figure 18:**
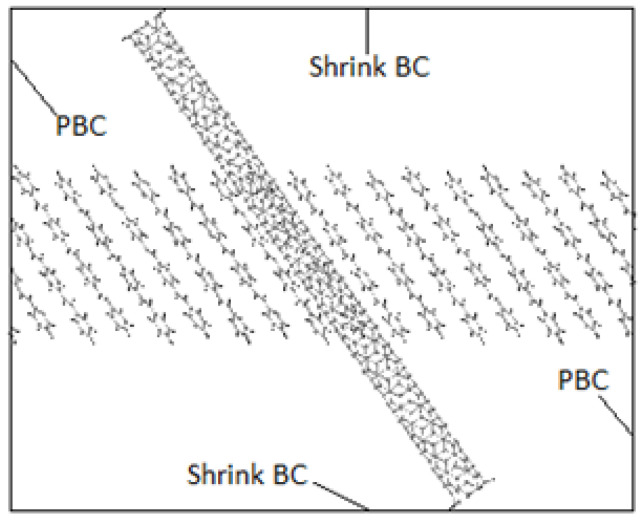
Boundary conditions of simulated PPV with embedded SWCNT.

In order to study the interface, CNMs are kept fixed by setting the force during each step to zero. This eliminated the interaction of responses of the two systems (CNM and crystal) and allowed the pure behavior of the polymer crystal with respect to the CNMs to be identified. Moreover, it has been reported by Yang et al. [62] that fixing the CNM reduces computation time. A validation of this procedure was confirmed through simulating the interaction 5PPV with fixed and free SWCNT. The difference in the potential energy of the systems was found to be smaller than 1%. The 4PPV HB crystal was interfaced with 6 nm×6 nm and 7 nm×7 nm graphene sheets, with coverages of 0.3653 (36.53%) and 0.4972 (49.72%). Perfect alignment between the sheet and 4PPV crystal was not crucial as multiple interfacial orientations occur in practice (Figure 17). Additionally, a SWCNT was embedded into a supercell of PPV crystal where the periodic and shrink boundaries were maintained as shown in Figure 18.

**Figure 19:**
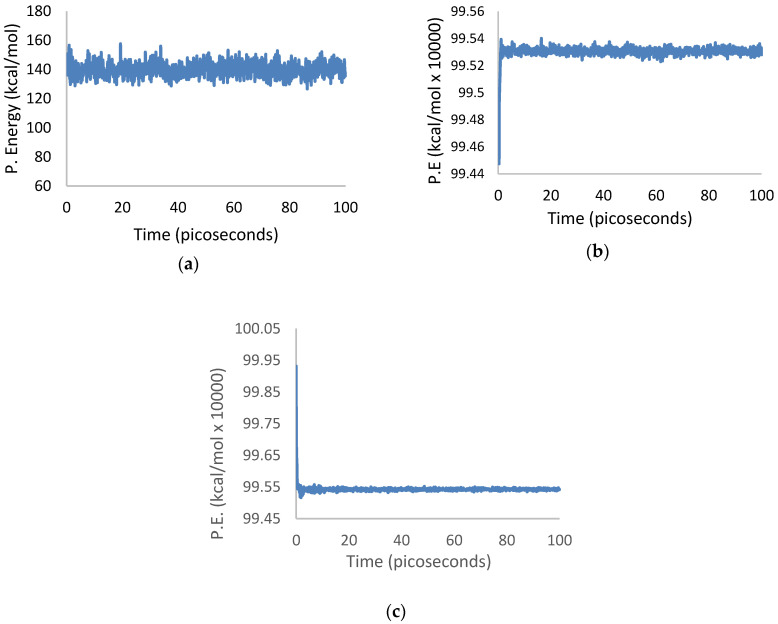
Potential energy equilibration over simulation time of 3PPV and (13,0) SWCNT systems: (**a**) Potential energy vs. time of isolated 3PPV; (**b**) potential energy vs. time of isolated (13,0) SWCNT; (**c**) potential energy vs. time of composite 3PPV–(13,0) SWCNT.

The potential energies of isolated 3PPV, isolated (13,0) SWCNT, and 3PPV/(13,0) SWCNT systems, are presented in Figure 19a–c. All plots show the systems to be equilibrated within the first 10 ps of simulation, which occurred much earlier than the ensemble sampled times between 50 ps to 100 ps. Similar equilibration patterns are evident in the temperature versus time plots of the three systems.

**Figure 20:**
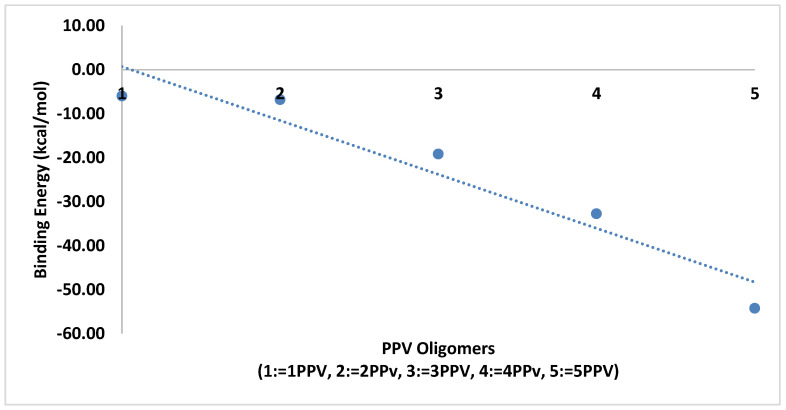
Binding energy PPV oligomers–graphene.

The average binding energies of the oligomers are higher with graphene as compared to SWCNTs. This is because the surface area for binding is maximized for better π−π interactions. The reported binding energy of 3PPV–graphene, ≈−24 kcal/mol (Figure 20), is in good agreement with the values reported by Yaya et al. [63] for 3PPV–graphene (−24.67478 kcal/mol (−1.07 eV)).

**Figure 21:**
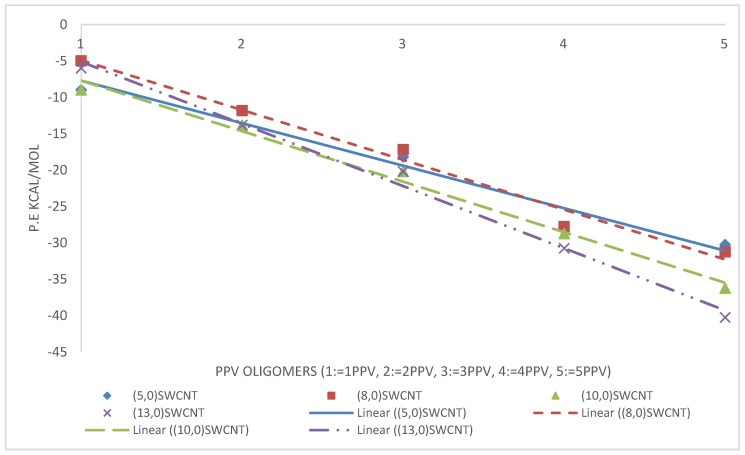
Binding energy of PPV oligomers with respect to (w.r.t) semiconductor SWCNTs.

**Figure 22:**
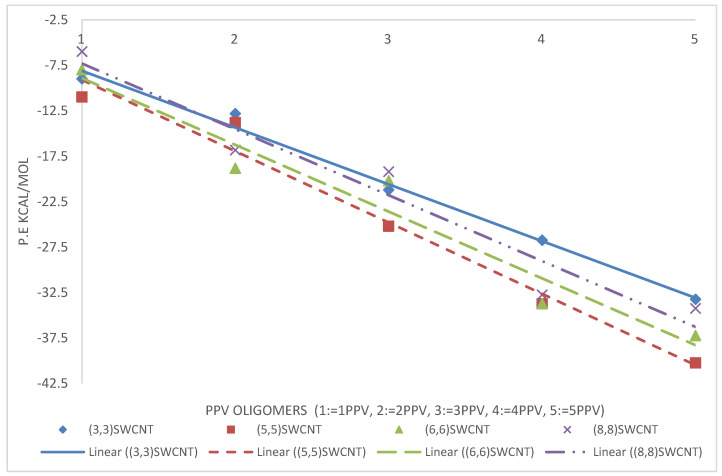
Binding energies of PPV oligomers w.r.t metallic SWCNT.

From the simulations, calculated binding energies for 3PPV-(13,0), 3PPV-(10,0), 3PPV-(8,0), and 3PPV-(5,0) were 22.5 kcal/mol, 22 kcal/mol, 18 kcal/mol, and 19 kcal/mol, respectively, and compared well to the obtained results from Yaya et al., [63] for “axial-parallel” configuration between 3PPV and SWCNT (see Figure 21 and Table 5). Moreover, the calculated binding energies of (8,8) and (6,6) metallic SWCNT to 3PPV were ∼22 kcal/mol and ∼23 kcal/mol, which are in good agreement with reported values from first-principle studies by Yaya et al., [63] for 3PPV-(6,6) in three different configurations with SWCNTs and 3PPV-(8,8), 3PPV-(4,4) for “axial-parallel” configuration. Furthermore, the calculated binding energies for 3PPV-(5,5) and 3PPV-(3,3) were ∼ 25 kcal/mol and ∼ 21 kcal/mol, respectively. These values were relatively higher (see Figure 22 and Table 6).

### 3.4. Structural Configurations

#### 3.4.1. PPV/Graphene

**Figure 23:**
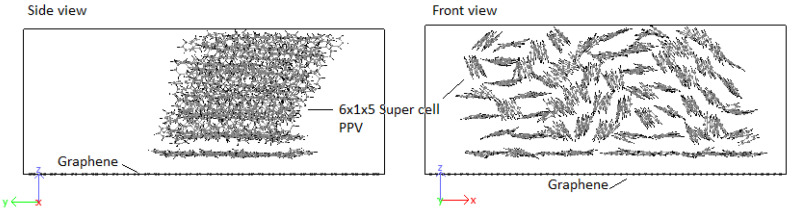
Interaction: 4PPV–7 nm × 7 nm graphene (final).

An initial 8 × 1 × 8 supercell of 4PPV is generated. Upon removal of wrapped molecules, a 6 × 1 × 5 supercell remains. This is interfaced with 6 nm×6 nm and 7 nm×7 nm graphene sheets, with coverages of 0.3653 (36.53%) and 0.4972 (49.72%). As the results for both graphenes are practically identical, final configuration, radial distribution function (RDF) and radius of gyration plots for HB PPV–7 nm×7 nm graphene are presented. According to Figure 23, on the interaction with graphene, PPV molecules nearest to the sheet orient perpendicular to the surface and after the adsorption, they reoriented to lie parallel to the sheet.

**Figure 24:**
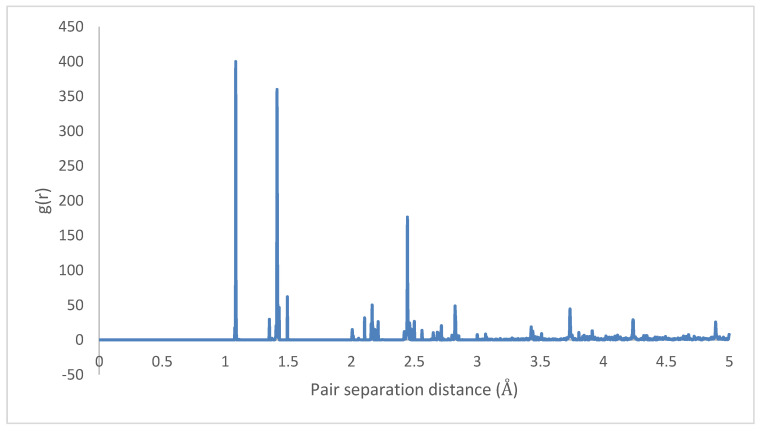
Radial distribution function: HB-4PPV–7 nm × 7 nm graphene (initial).

**Figure 25:**
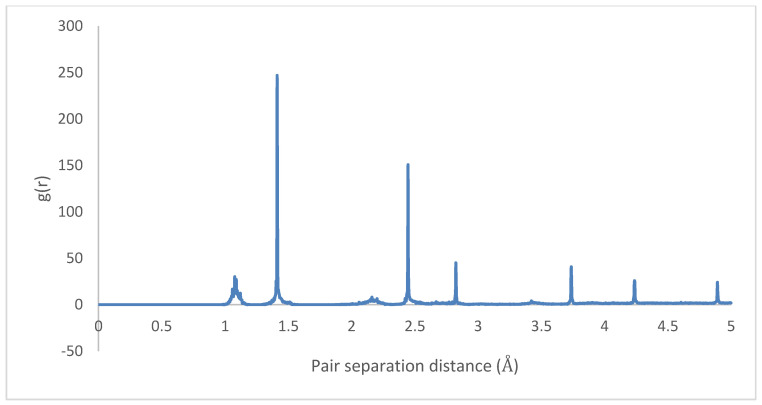
Radial distribution function: 4PPV–7 nm × 7 nm graphene (final).

The radial distributions for the initial and final states are depicted in Figure 23 and Figure 24, showing highly organized systems. Figure 24 represents a highly crystalline HB structure, while Figure 25 shows that the crystalline system becomes more diffused than the HB arrangement but not strictly amorphous, for example, the coarsely arranged brick-wall conformation.

**Figure 26:**
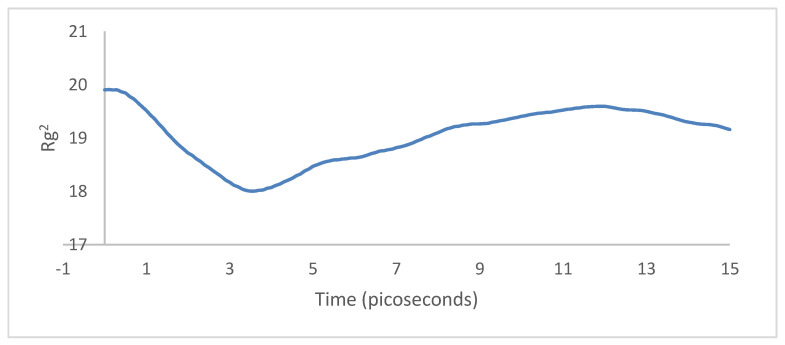
Radius of gyration: 4PPV–7 nm × 7 nm graphene).

The radius of gyration profile over the simulation period, as shown in Figure 26, dropped from about 20 to 18. The supercell initially contracts and then spreads onto the sheet surface after about 3 ps. This was confirmed by the trajectories of the molecules. The net difference between the initial and final configurations was  ∼−0.8, which indicates that the system is more compact than its initial state. Hence, there is more binding energy between the systems.

#### 3.4.2. PPV/SWCNT

**Figure 27:**
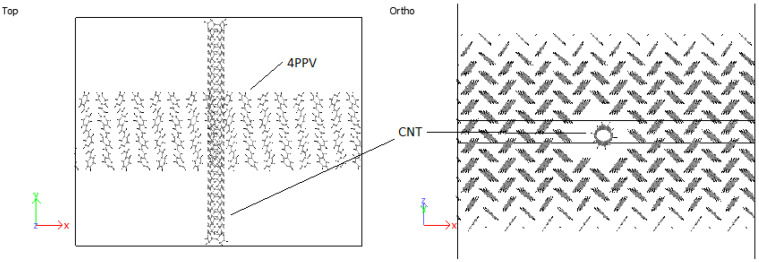
Interaction: 4PPV-SWCNT (initial).

**Figure 28:**
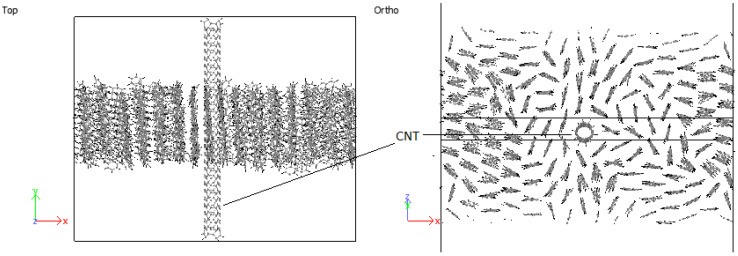
Interaction: 4PPV-SWCNT (final).

In all cases considered for embedded SWCNTs into PPV crystals, PPV molecules were found to be adsorbed onto the walls of the carbon nanotube (CNT). The PPV tried to arrange its rings’ surfaces parallel to the walls of the CNT. There were space confinements due to the bulk, and therefore not all PPV could lie flat. In the periodic supercell of 8 × 1 × 8 PPV, all HB structures were rearranged to the brick-wall structure within the simulation time demonstrated in Figure 27 and Figure 28.

**Figure 29:**
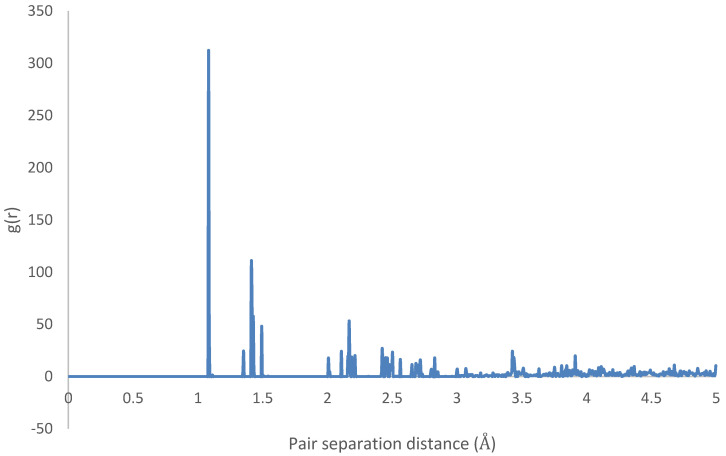
Radial distribution function (RDF) of HB-4PPV with carbon nanotube CNT (initial).

**Figure 30:**
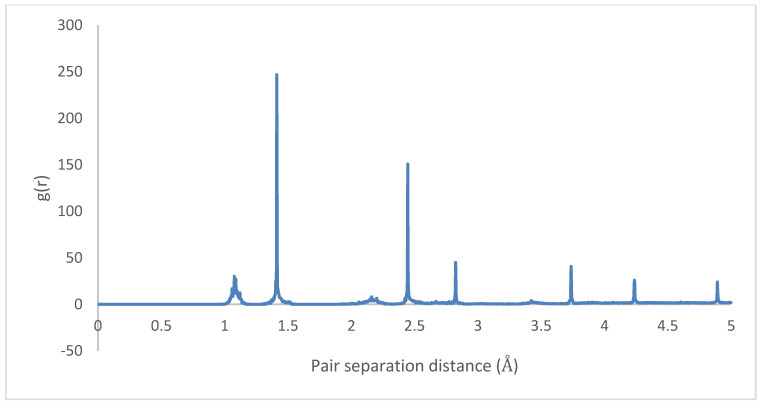
Radial distribution function (RDF) 4PPV with CNT (final).

Therefore, it can be inferred that CNT-PPV interactions are primarily π−π dominated, as it is evident in the rearrangement of PPV molecules to align flat with CNM surfaces. The interactions propagate significantly through nearby lattices, forcing them to take on the brick-wall configuration, which allows optimal interactions of the π-bonds with the molecules. The same trends were observed for both zigzag and armchair CNTs, as well as CNTs with larger diameters. The RDF plots show the change in morphology of the PPV supercell, before and after interacting with the SWCNT (Figure 29 and Figure 30). The initial RDF clearly shows a highly crystalline supercell. This is characterized by the lack of diffused states between occurring peaks. The presence of the CNT disrupts the order of the supercell, and the disorder is propagated through the supercell. Consequently, there are more diffused states (brick-wall) present.

**Figure 31:**
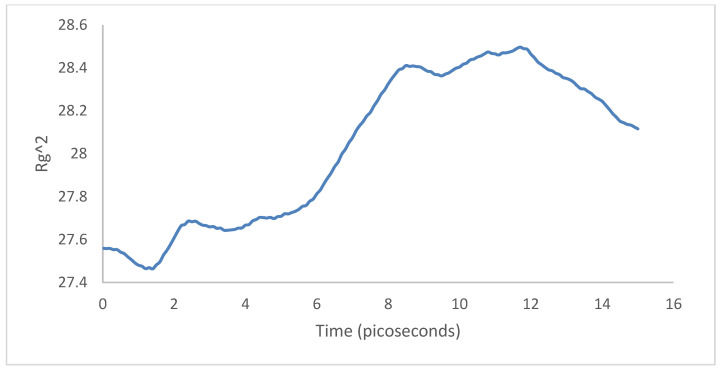
Radius of gyration: 4PPV–CNT.

For the case of the radius of gyration, first the PPV supercell contracted and then expanded around the SWCNT. There was a net difference of ∼+0.6 in the radius of gyration between the initial and final configurations (Figure 31), indicating that the system was more spread out than it initially started. Hence, there was less interfacial energy after the simulation.

## 4. Discussion

### 4.1. Geometry Optimization

Optimized bond lengths and angles are in good agreement with theoretical and experimental results obtained from literature. $LJ_{B3LYP}$ and $LJ_{AUTODOCK}$ parameters capture the stable C-H…π HB crystalline arrangement for PPV, the former, $\sigma_{B3\mathrm{LYP}}$ and $\epsilon_{B3\mathrm{LYP}}$ was used. The reason being that the QM/MM generated LJ parameters capture H geometries and bond energies more properly and have good agreement with experimental results, and have better accuracy in predicting intermolecular interaction as it has been obtained by studying over 100 independent bimolecular organic systems [59].

### 4.2. Crystallization

Achieving the HB conformation is highly dependent on the starting simulation configuration. This together with the correctly chosen LJ-parameters will lead to the correct crystal structures. It should be noted that, due to elevated temperatures of the annealing process, all oligomers displayed larger chain torsions at higher temperatures. The initially assigned unit cell dimensions [9], see Section 4.2, were comparable to experimentally obtained results by [64,65,66,67] with an average density $\rho =1.06404{\;g\;\mathrm{cm}}^{-3}$.

Due to the fluctuation in temperature in the final equilibration phase, a constant setting angle could not be determined. Rather, the setting angle, $\phi_{s}$, fluctuated approximately between $50^{{}^{\circ}}-70^{{}^{\circ}}$. A cursory look at the trajectories of adjacent molecules show that $\phi_{s}$ varied due to the relative motion of phenyl rings of one chain to that of the other, and consequently, as well the relative location of the vinyl links with respect to each other on the chains. The chains re-order alongside each other in the direction of the longitudinal molecular axis. Accordingly, $\phi_{s}$ is highest when adjacent phenyl rings coincide in their motions, and lowest when the phenyl ring and vinyl link of adjacent chains coincide. The $\phi_{s}$ range compares to the range given by Granier et al., [68] of approximately $56^{{}^{\circ}}-68^{{}^{\circ}}$.

### 4.3. Intramolecular Interactions and Binding Energy

As expected, the potential energy of 1PPV, 2PPV, 3PPV, 4PPV and 5PPV, increased linearly by approximately 50 kcal/mol, corresponding directly to the increase in consecutive chains by one monomer unit each. Metallic SWCNTs (M-SWCNTs) have slightly more potential energy than semiconducting SWCNTs (SC-SWCNTs), this was also to be expected as the diameters of metallic SWCNT are fractionally larger, and hence they contain more atoms.

Overall, the binding energy between oligomers and SWCNTs increased by diameter. This is due to the fact that increasing diameter increases the surface area for binding of the SWCNT, which allows for better π−π interactions between the molecules and SWCNTs.

### 4.4. Structural Configurations

Crystal PPV interfacing with SWCNTs and graphene reorganizes the crystal at the interface to optimize π–π interactions at 300 K. The interactions are stronger with graphene than SWCNTs due to its larger effective interfacing surface area. These results are highly localized due to the scale of the simulation, hence limiting the viability of these findings to the nano regimes.

## 5. Conclusions

In the current study, initial binding energies of several PPV oligomers (1PPV to 5PPV) were predicted using molecular dynamic simulations at 300 K. It was found that the number of molecules in the unit cell, and the unit cell dimensions are of importance to arrive at the possible conformations, and avoid the system being trapped in local minima. Moreover, it was observed that in achieving HB-structure in oligo-PPV, the Lennard-Jones parameter was crucial. This comparative study suggests that the Lennard-Jones parameters calculated by Freindorf et al. [59] for $LJ_{B3LYP}$ is the best choice. The binding energies of oligo-PPV to CNMs increased linearly with chain length, and SWCNT diameter. Furthermore, the results confirm that the most stable configuration of pristine PPV at 300 K was the herringbone arrangement, and oligo-PPV with herringbone structure reoriented to maximize the π–π interactions with the CNMs’ surfaces. Finally, radial distribution profiles, together with the radius of gyration profiles indicate that HB-PPV interaction with graphene was much stronger than with embedded SWCNTs.

## Figures and Tables

**Table 1:** Geometrical parameters of single-walled carbon nanotubes (SWCNTs).

Approximate Diameter (Å)	Metallic SWCNTs				Semiconductor SWCNTs			
	n	m	Actual Diameter (Å)	Length (Å)	n	m	Actual Diameter (Å)	Length (Å)
4	3	3	4.01	71.4	5	0	3.98	72.5
6	5	5	6.75	71.4	8	0	6.27	72.5
8	6	6	8.14	71.4	10	0	7.83	72.5
10	8	8	10.85	71.4	13	0	10.11	72.5

**Table 2:** A comparison of PPV geometry optimization obtained in this study and cited works.

Parameters	UFF Stilbene (This Work)	UFF 3PPV (This Work)	Isolated Chain [9]	Isolated Chain [54]	Experiment [55]	Styrene [9]
Bond Lengths (Angstroms)						
C2–C4	1.398	1.398	1.375	1.383	1.387	1.381
C1–C2	1.398	1.398	1.401	1.411	1.397	1.384
C1–C3	1.398	1.398	1.403	1.414	1.394	1.395
C6–C7	1.464	1.467	1.451	1.443	1.469	1.449
C7–C8	1.323	1.323	1.349	1.361	1.318	1.329
C2–H	1.085	1.085	1.096	1.103	0.93	1.095
C3–H	1.085	1.085	1.096	1.104	1.02	1.096
C7–H	1.085	1.085	1.101	1.108	1.00	1.098
**Bond Angles (Degrees)**						
C2–C1–C3	120.0	120.0	116.7	117.3	117.8	119.6
C1–C2–C4	120.0	120.0	120.9	121.1	121.4	119.7
C1–C3–C5	120.0	120.0	122.4	121.6	120.6	120.7
C6–C7–C8	119.7	119.6	127	126.3	126.7	128
C1–C2–H	119.9	119.9	119.9	119.4	117.2	120.2
C1–C3–H	119.9	119.9	117.9	118.7	119	119.6
C6–C7–H	120.8	120.8	114.6	115	116.1	113.5

**Table 3:** Lennard Jones σ and ϵ values for (a) theoretically and (b) experimentally obtained parameters.

(a) THEORETICAL								
	B3LYP/6-31+G*/AMBER [59]		AUTODOCK [60]		HF/3-21G/TIP3P [58]		AM1/TIP3P [57]	
	Sigma	Epsilon	Sigma	Epsilon	Sigma	Epsilon	Sigma	Epsilon
C…C	4.02	0.06	4.00	0.150	3.80	0.08	3.50	0.08
H…H	2.22	0.03	2.00	0.020	2.60	0.01	2.00	0.01
C…H	3.12	0.04243	3	0.05477	3.2	0.02828	2.75	0.02828
**(b) EXPERIMENTAL**								
	**AMBER-94 [56]**				**UFF [47]**			
	Sigma		Epsilon		Sigma		Epsilon	
C…C	3.40		0.10		3.851		0.105	
H…H	2.53		0.02		2.886		0.044	
C…H	2.965		0.04472		3.3685		0.06797	

**Table 4:** Initial simulation cell for oligomer crystallizations.

PPV	a=Δx	b=Δz	c=Δy
Monomer	8.07 Å	6.05 Å	6.54 Å
2PPV [55]	12.381 Å	5.723 Å	15.71 Å
3PPV [61]	8.1 Å	6.05 Å	24.55 Å
4PPV	9.5 Å	6.0 Å	31.4 Å
5PPV [26]	8.58 Å	6.13 Å	39.14 Å

**Table 5:** Binding energies of 3PPV w.r.t semiconductor SWCNT.

This Work	This Work	Yaya et al. [63]
3PPV-(13,0)	~−22.5 kcal/mol	−22.3687 kcal/mol (−0.97 eV)
3PPV-(11,0)	----	−21.6769 kcal/mol (−0.94 eV)
3PPV-(10,0)	~−22 kcal/mol	----
3PPV-(9,0)	----	−21.4463 kcal/mol (−0.93 eV)
3PPV-(8,0)	~−18 kcal/mol	----
3PPV-(7,0)	----	−20.0627 kcal/mol (−0.87 eV)
3PPV-(5,0)	~−19 kcal/mol	----

**Table 6:** Binding energies of PPV oligomers w.r.t metallic SWCNT.

This Work	This Work	Yaya et al. [63]
3PPV-(10,10)	----	−23.2911 kcal/mol (−1.01 eV)
3PPV-(8,8)	~−22 kcal/mol	−22.3687 kcal/mol (−0.97 eV)
3PPV-(6,6)	~−23 kcal/mol	−21.4463 kcal/mol (−0.93 eV)
3PPV-(5,5)	~−25 kcal/mol	----
3PPV-(4,4)	----	−20.5239 kcal/mol (−0.89 eV)
3PPV-(3,3)	~−21 kcal/mol	----
